# The *Bionic Radiologist*: avoiding blurry pictures and providing greater insights

**DOI:** 10.1038/s41746-019-0142-9

**Published:** 2019-07-09

**Authors:** Marc Dewey, Uta Wilkens

**Affiliations:** 1 Charité—Universitätsmedizin Berlin and Berlin Institute of Health, Berlin, Germany; 2 Ruhr-University Bochum, Institute of Work Science, Bochum, Germany

**Keywords:** Medical imaging, Society

## Abstract

Radiology images and reports have long been digitalized. However, the potential of the more than 3.6 billion radiology examinations performed annually worldwide has largely gone unused in the effort to digitally transform health care. The *Bionic Radiologist* is a concept that combines humanity and digitalization for better health care integration of radiology. At a practical level, this concept will achieve critical goals: (1) testing decisions being made scientifically on the basis of disease probabilities and patient preferences; (2) image analysis done consistently at any time and at any site; and (3) treatment suggestions that are closely linked to imaging results and are seamlessly integrated with other information. The *Bionic Radiologist* will thus help avoiding missed care opportunities, will provide continuous learning in the work process, and will also allow more time for radiologists’ primary roles: interacting with patients and referring physicians. To achieve that potential, one has to cope with many implementation barriers at both the individual and institutional levels. These include: reluctance to delegate decision making, a possible decrease in image interpretation knowledge and the perception that patient safety and trust are at stake. To facilitate implementation of the *Bionic Radiologist* the following will be helpful: uncertainty quantifications for suggestions, shared decision making, changes in organizational culture and leadership style, maintained expertise through continuous learning systems for training, and role development of the involved experts. With the support of the *Bionic Radiologist*, disparities are reduced and the delivery of care is provided in a humane and personalized fashion.

“For almost all conclusions the degree of their probability should be determined, if possible expressed in numbers.”

- Eugen Bleuler, Autistic-undisciplined thinking in medicine and overcoming it, 1922^1^

## Introduction

### Economic landscape

**Fig. 1:**
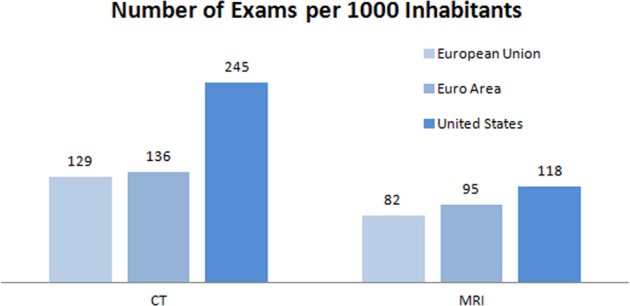
Number of CT and MRI examinations per 1000 inhabitants for the European Union, the Euro Area, and the United States. Data are shown for those countries in the European Union and Euro Area for which total numbers of CT (computed tomography) and MRI (magnetic resonance imaging) exams were available, i.e. the sum of in- and outpatient data. None of the European Union countries had higher utilization than the United States. Data sources: Barmer GEK Arztreport 2011 (for data from Germany). OECD (2018), Computed tomography (CT) and Magnetic resonance imaging (MRI) exams (indicator). 10.1787/3c994537-en and 10.1787/1d89353f-en

From an economic perspective, the inappropriate use and overuse of diagnostic testing is important as it accounts for a substantial portion of the $340 billion reported annually for all unnecessary or inefficiently delivered services in the United States.^2^ Moreover, the higher utilization of expensive imaging procedures in the United States is striking when compared with the European Union and the Euro Area (Fig. 1). This difference is possibly driven by the availability of universal health coverage systems in Europe,^3^ which has been shown to lead to highest equity, access, quality, and resource savings.^4^ Nevertheless, values that matter most to patients, such as quality of life and avoiding complications,^5^ have become secondary at best in most health care systems around the world and economic considerations instead of physician ethos are driving medicine.^6,7^

### Problem formulation

Beyond the economic forces driving medicine into directions that are not directed at benefiting patients first, there are three major self-made problems in diagnostic imaging that warrant consideration. First, clinical service lines rarely calculate degrees of disease probabilities of individual patients before diagnostic decision making. Second, radiology images of the same body part are often inconsistently analyzed even within a health care center.^8^ Third, reports of radiology findings often lack adequate structure and frequently use idiosyncratic terminology^9^ that does not match with data needed for personalized treatment decisions resulting in missed care opportunities as shown for reported aortic aneurysms.^10^

### Value-based radiology

The goal of value-based medicine is to improve patient outcomes while at the same time reducing cost.^11^ To be successful in supporting the implementation of value-based medicine,^12^ value-based radiology needs to ensure integration of diverse sources of information and patient centeredness.^13^ Value-based radiology will also assume a central role in solving the above economic problems, with attention to the three problem formulated above: (1) Improving decision making about when to obtain diagnostic imaging tests, (2) Increasing consistency in image analysis, and (3) Enhancing the link between test results and treatment recommendations (Box 1).^14^ To be successful in these three areas, the *Bionic Radiologist*^14^ needs to be established in order to make judicious use of advances in artificial intelligence and digital medicine in radiology.^15^

### Vision of the future

Wouldn’t it be great if patient decisions about diagnostic testing were influenced by degrees of disease probabilities and patient preferences, if radiology images would be analyzed consistently at any time of the day and at all sites, and if treatment recommendations were closely linked to radiology imaging results building on high physicians’ trust in and commitment to such new processes to avoid missed care opportunities? The vision is thus to avoid blurry pictures and provide greater insights for treatment recommendations by the *Bionic Radiologist*.

Box 1: aims of the *Bionic Radiologist* - Guiding decisions about which, if any at all, imaging test should be done in individual patients

- Augmenting human perception and interpretation tasks of image findings analysis

- Facilitating treatment suggestions by integrating all available evidence with imaging results

## Towards the *Bionic Radiologist* from a socio–technical perspective

The *Bionic Radiologist* is an approach to radiology combining machine intelligence,^16–18^ developed using well-curated medical data,^19^ and a human radiologist to leverage both the consistency of automatic analysis and individual nuances of human perception and interpretation.^14,20^ This approach is similar to the integration of technology and humanisms portrayed by Dr Leonard McCoy in Star Trek.^21,22^ This convergence of human and artificial intelligence and its relevance for high-performance medicine has recently been described in detail by Eric J. Topol.^23^ From an overall system development perspective it is crucial that the integration of machine intelligence systems with human radiologists leads to an advanced human-computer-interaction with new forms of collaboration^24^ enhancing the expertise in diagnosis and decision making,^25^ while individual reservation and resistance to collaborate with computers in decision making^26^ needs to be considered as an implementation barrier (Table 1). There are certain models providing a deeper understanding of resistance to change in technology-intensive work environments. The technology acceptance model from Davis^27^ argues that technology acceptance is a matter of the perceived usefulness of a technology and its ease of use. This implies that the black-box nature of artificial intelligence solutions need to be overcome as they are perceived as having an opaque design and limited transparency of decisions.^28^ To increase the chance that the integration of machine and human intelligence works, automated suggestions should become understandable for instance using uncertainty quantification.^29^ Transparency and clear decision structure can be estimated as a necessary prerequisites for technology acceptance but they do not define sufficient conditions. The social acceptance of new technologies, individual behavior and attitudes need to be integrated in the conceptual outline.^30^ In this regard the consideration of perceived justice with the respect to the physicians’ expert role^31^ is an issue. Wilkens & Artinger^25^ emphasize the mindfulness of individual expertise in workplaces with distributed intelligence in order to explain counterproductive work behavior respectively pro-active use of technologies. Another crucial issue is the learning scenario which is provided as part of the socio–technical system design. The radiology AI tools, described in detail below in the impact section, provide new opportunities for continuous learning systems that help in training radiologists and radiology residents and integrating the advancement of medical proficiency. But at the same time physicians need to know how to feed back observations, critical remarks, potential solutions etc. to the overall system. With respect to social acceptance it is important to avoid conditions under which individuals continuously learn from machines but where it is not clear how machines learn from individuals. A mutual learning process between machine and the human beings can be considered as indicator for advancements in the social–technical work system.

**Table 1:** Implementation barriers for the *Bionic Radiologist* and possible solutions

Implementation barriers	Possible solutions
Reluctance to delegate physician decision making, even if only in part, to black-box systems	Uncertainty quantification for such decision making to increase transparency of predictions Advanced human-computer-interaction enhancing the expertise in diagnosis and decision making
Poor work satisfaction of physicians because of an apparently reduced decision making role	Greater involvement in shared decision making with patients for increased work satisfaction Acceptance beyond technological usability e.g. through development of physicians roles
Expected decrease in image interpretation knowledge among physicians in the near future	Maintained expertise through automated feedback systems fed by patient events and outcomes Changes in culture and leadership style for enhancing physicians’ commitment
Perception that patient safety and trust are at stake by automated treatment suggestions	Human quality control and oversight of any treatment decisions put into effect Considering perceived justice in the care process through human-computer interaction

## Impact of the *Bionic Radiologist*

### Guiding decision making

The foremost impact of the *Bionic Radiologist* is getting the right imaging tests done, if any at all, in the right patients at the right point in time.^32^ The prevalent issue of too much imaging in the wrong patients could be overcome by better decisions about which patients should undergo a specific imaging procedure and when.^33^ This requires the integration of patient’s history into decision support modules of electronic clinical decision support systems. Such systems should be based on validated clinical prediction rules that generate individual probabilities of disease, before and after diagnostic tests according to Bayes theorem, and can thus improve decision making about referral for diagnostic imaging. Computer-based clinical decision support systems can increase appropriateness of imaging for several diagnostic imaging scenarios,^34^ and are also suitable for shared decision making incorporating individual patient values.^35^ Values that matter most to individual patients can thus be integrated into the decision whether or not to perform certain diagnostic imaging procedures and what to do with imaging test results based on treatment preferences expressed by patients. The recent eGUIDE initiative of the European Society of Radiology teaches medical students and clinical residents about the principles of estimating disease probabilities and the subsequent appropriate selection of diagnostic tests and thus has the potential to bring this part of the concept of the *Bionic Radiologist* to medical undergraduate curricula as well as radiology resident training (http://www.eurosafeimaging.org/esr-eguide).^36^

Moreover, providing diagnostic decision making tools, as well as automatically and human-labeled images and structured reports (see below), electronically to referring physicians and patients will facilitate more active involvement of patients in their own testing. This provides the opportunity for greater involvement of radiologists in shared decisions making with patients, which in turn is a solution in itself to avoid poor work satisfaction,^37^ because of otherwise reduced decision making roles (Table 1).

There are already at present several examples for guiding decision making about imaging tests with the help of other clinical information for instance from the medical records or laboratory tests.^34^ For instance, neural networks can be used to predict the probability of brain injuries in elderly patients presenting with head injury after a fall which may be used for guiding testing decision.^38^ Natural language processing of clinical information provided by referring physicians and demographic data can be used to predict the protocol and priority of brain MRI examinations.^39^ Clinical prediction models can provide probabilities for instance of pulmonary embolism which has shown to improve the yield of imaging and avoid imaging in those not needing it.^40^ It is also know that patients with stable chest pain and low-to-intermediate clinical probability of coronary artery disease (7–67%) benefit most from coronary CT angiography.^41^ This information can readily be integrated into decision making about further testing in patients presenting with chest pain. An important barrier to implementation of such decision support systems in clinical practice is the hesitation of physicians to refrain from diagnostic testing in patients who are actually suggested to not need further imaging tests.^42^ Another practical challenge is that the half-life of clinical data for training decision support systems is just four months indicating that most recent data are needed and continuous updating is crucial.^43^

### Augmenting human image analysis

A true augmented radiologists’ image analysis by machine intelligence may increase consistency and avoid perception and interpretation errors when reviewing patients’ radiology images.^15^ The potential of this becomes clear when noting that present-day artificial neural networks are as accurate as radiologists in detecting breast cancer on a mammogram.^44^ However, there are also more false positive findings using current deep learning image analysis techniques for instance for detection of critical findings in head CT,^45^ which may lead to radiologists’ fatigue posing a safety risk and should thus be avoided using better technology. The *Bionic Radiologist* as a combined approach to leverage both the consistency of automatic analysis and individual nuances of human image analysis would possibly have greater value for clinical practice. This however implies considerable changes in the work flow and will require a revolutionary shift in how radiology is practiced today: data science and artificial intelligence will be practically and physically integrated into the workplace of radiologists and interrelated functions. The *Bionic Radiologist* will be a radiologist supervising the results generated by machine learning algorithms and integrating them with other clinical data for the final interpretation.^14^ Interestingly, such a hybrid approach might also be most acceptable to patients according to a survey at Charité among 100 patients clinically referred to computed tomography; 85% preferred an analysis by radiologists and computers compared to either of them alone.

The *Bionic Radiologist* approach would be similar to the situation in the airplane cockpit where the autoflight system is used most of the time, but for the situations in which human interaction cannot be replaced one prefers to have a pilot on board. Interestingly, the human pilot can better control for anomalies than the autopilot^46^ and shared control architecture should thus be considered for medical use. We also need to continue to advance the currently rather basic information technology infrastructure in health care so that it can integrate multifaceted information from all disciplines including radiology.^47^ This will allow radiologists more time for what should be their two primary roles in addition to interpreting images: talking empathetically to patients who are undergoing procedures and consulting with referring physicians. In general, this effect has potential to revitalize the patient-physician relationship. However, if the role shifts toward only supervising results generated by artificially intelligence a decrease in image perception and interpretation skills among physicians has to be expected to occur in the future. Expertise should thus be maintained through training that includes automated and individualized feedback systems to the human reader about his or her cases which is fed by patient events and outcomes (Table 1). Also, the training of radiology residents according to structured approaches such as the European Training Assessment Program needs to include teaching future radiologists about artificial intelligence and specifically augmented image interpretation. Moreover, gaming approaches to image analysis and randomly selected comparisons of pure human reads with augmented reads by the *Bionic Radiologist* can keep motivation high and may even improve skills be increasing the knowledge base and feedback mechanisms. Digital competencies are an important prerequisite to make use of new technologies for better and innovative solutions.^48^ A participatory process development is inevitable for enhancing physicians’ commitment and successful implementation can also imply changes in institutional culture and leadership style.^49^

There are already at present examples for augmenting human image analysis in radiology. These are mostly based on deep learning yet also include texture analysis using radiomics^50^ or fractal analysis.^51^ In brief, deep learning has been successfully applied to several fields using convolutional neural networks: scoring knee osteoarthritis on radiographs,^52^ improving detection of wrist fractures on radiographs,^53^ detecting breast cancer on mammograms,^44^ augmenting the ability of radiologists to detect cancer on screening mammograms without increased reading times,^54^ classifying breast masses on ultrasound,^55,56^ prioritizing the worklist of radiologists for suspected intracranial bleeding on CT,^57^ detecting and possibly sub-classifying intracranial bleeding on CT,^58^ identifying critical findings on head CT with somewhat limited sensitivity for stroke,^59^ predicting ischemic stroke onset time on MR imaging,^60^ classifying six common liver tumor entities,^61^ triaging chest radiographs for urgent findings,^62^ pulmonary nodule detection on chest radiographs,^63^ and predicting 2-year survival in patients with non-small cell lung cancer based on standard-of-care CT images of the chest.^64^

Texture analysis from radiomics may be used independently or as an input for deep learning. A combination of radiomics features and a convolutional neural network can for instance be used for identifying complete response of muscle-invasive bladder cancer on CT imaging^65^ and coronary flow reserve can be estimated based on CT angiography data using machine learning.^66^ Moreover, bone age assessment based on radiographs of the hand can be automated using convolutional neural networks that result in greater consistency than human reads^67^ and a recent challenge showed consistent results with small mean age differences when convolutional neural networks were applied for this task.^68^ Fractal analysis allows to characterize the underlying nature of the vascular tree in cancer grading^51^ and also myocardial perfusion assessment.^69^ As fractal analysis is inspired by pathophysiological changes it may also function well as an input for deep learning or for deriving quantitative maps of perfusion for human or augmented interpretation.

### Facilitating treatment suggestions

The *Bionic Radiologist* will also be the authority in developing and realizing structured radiology reports.^9^ Structured radiology reports are searchable and quantifiable descriptions of radiology images for all different kinds of imaging tests for instance of tumors or abnormalities in the cardiovascular system.^70,71^ Such structured reports maximize objectivity and reduce variability of prose text most often used in reports and enhancing the link between radiology findings and treatment suggestions.^72^ Structured radiology reports may either be automatically generated from unstructured human radiology reports using advanced language processing,^73,74^ which does not require any cultural changes in clinical radiologic practice at the institutions, or they could be achieved by the more difficult cultural transition to structured reporting by radiologists. Structured reports generated by the *Bionic Radiologist* described above would also facilitate the linkage of the results to the wording of evidence-based and personalized treatment recommendations. The integration of evidence-based recommendations facilitated by data sharing^75^ to radiology results reporting would certainly be a sweet spot for the use of data and computer science developments as nowadays the doctors, despite sub-specialization, cannot keep track of the volume of the relevant literature published for instance about cardiac imaging.^76^ To become successful at scale, integration requires collaborations such as the American College of Radiology’s Data Science Institute (www.acrdsi.org) as well as efforts leading to more widespread use of quantitative imaging biomarkers such as the Quantitative Imaging Biomarkers Alliance (www.rsna.org/QIBA) and global health data sharing from pivotal clinical trials in radiology.^75^

Structured reporting will thus transform the culture of radiology reporting practice from prose text documents towards structured reports which have greater standardization and enable searchability. This requires acceptance on the human side for changes in the workplace which affect the culture of radiology as a clinical discipline. This includes overcoming the perception that patient safety and trust may be at stake if treatment recommendations are automatically generated by algorithms based on integration of imaging findings with other clinical information using computer-led systems.^77^ This can be addressed—in analogy to the interplay between human pilot and autopilot—by e.g. a greater role of humans in quality control and oversight of any treatment decisions before they are put into effect (Table 1). This greater involvement in the entire process of patient care from diagnostic and prognostic to therapeutic tasks will become possible by avoiding physicians having to perform repetitive and mundane tasks.^78^ Job design and job development have to face the criteria which are related to such a role development and open mindset for new forms of human-computer collaboration.^31^

Several examples of structured reporting for improved linking of imaging findings with treatment suggestions exist. For instance, structured reporting of multiphasic CT of pancreatic cancer improves the completeness of reports while structured rectal cancer MR imaging reports reduce the need for further treatment planning consultations.^72^ Similar electronic solutions are available for breast,^79^ coronary artery,^80^ and prostate imaging. There are also IT solutions that standardize radiology reports of abdominal aortic aneurysms and facilitate communication of results and patient follow-up.^81^ Importantly, natural language processing methods now allow to automatically extract and characterize the clinical significance of findings in radiology reports^82^ and convolutional neural networks have better accuracy for radiology text report classification than rule-based models,^83^ which holds potential to facilitate linking treatment recommendations with findings provided that a sufficient level of standardization is present in reports.^73^

## Implementation of the *bionic radiologist*

**Fig. 2:**
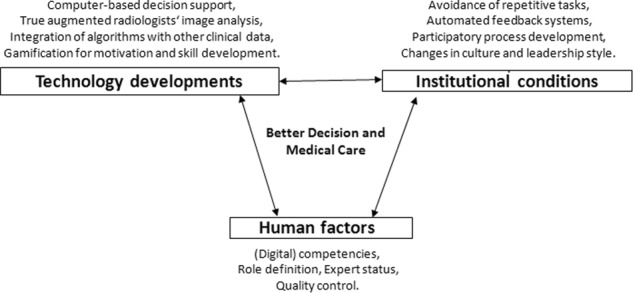
The *Bionic Radiologist* from a socio–technical system perspective and recommendations for implementation. Characteristics and facilitating factors for the *Bionic Radiologist* with respect to the technology development itself, the surrounding institutional conditions and resistance to cultural change as well as the prerequisites for individual human acceptance and adaptation

Enabling better decisions by the *Bionic Radiologist* about whether or not to do an imaging test depends on a consistently obtained patient history and clinical background, clinical prediction rules, and models for decision making. To implement this integration of human and machine decision making in medical care requires uncertainty quantifications for decisions and predictions in order to obtain acceptance by the human radiologists. Integration of artificial and individual intelligence will also benefit from the digital transformation as a socio–technical system with dynamics that balance interests on all sides.^84^ Using the opportunities of artificial intelligence for image analysis in clinical practice requires a seamless integration with human image analysis. This will also provide the radiologist with greater freedom for involvement in shared decision making with patients which will increase work satisfaction.^85^ This is in line with a recent survey of radiologists who expect an increase in the time spent with patients as a result of artificial intelligence implementations as well as a lower risk of imaging-related medical errors.^86^ Acceptance by human radiologists will be further increased by automated and individualized feedback systems to the human readers about outcomes of his or her patients and thus enhancement of the expert role. *Bionic radiologists* will use structured reports that include automatically generated reminders about imaging findings that require follow-up as well as treatment recommendations to avoid missed care opportunities and increase patient safety and trust of all stakeholders involved in health care by oversight of treatment decisions. The overall goal is to provide better integrated health care through the *Bionic Radiologist* combining digitalization with human input at all stages. Success in the implementation will ensure that advances in artificial intelligence and digital medicine benefit diagnostic management decisions by providing calculated disease probabilities to avoid disparities and provide humane and personalized health care universally. The implementation of the *Bionic Radiologist* depends on the interplay between the new technological potential, individual behavior and institutional organizational properties whether the positive outcome in terms of better decisions and medical care can be attained.^87,88^ The most critical influencing factors are summarized in Fig. 2.

## Conclusion and outlook

The *Bionic Radiologists* promises better outcomes and lower costs through better integration of diagnostic imaging in clinical care processes. In this perspective we outlined the characteristics and facilitating factors with respect to the technology development itself, the surrounding institutional conditions and resistance due to the required change in culture as well as the prerequisites for individual acceptance and adaptation (see Fig. 2). Further advancements imply a socio–technical system development where these factors are related to each other in a fruitful and productive manner overcoming socio-cultural barriers to deployment of the AI augmented radiology and creating a win-win constellation for all involved stakeholders such as patients, physicians and cost-keepers in healthcare.

It is an issue of future research to further analyze what the interaction between these fields can look like and what the specific characteristics of relevant variables are for gaining better outcomes in terms of appropriateness, completeness, and timeliness. A longitudinal socio–technical system analysis with time-series analysis of health care data which are related to the characteristics of the workplace is supposed to be a suitable approach. This also implies that medical technology research could and should be further aligned to organizational studies and that the question of trust in AI-assisted medical imaging should play a central role in future research.
